# Research on the Structure Design and Mechanical Properties of Performance Optimized Multi-Axial Geogrid

**DOI:** 10.3390/polym14224939

**Published:** 2022-11-15

**Authors:** Zhiyuan Si, Xinhai Zhao, Xinbo Ren, Chao Zheng, Hongbo Yuan, Ji Liu, Libin Song

**Affiliations:** Key Laboratory of Liquid-Solid Structural Evolution & Processing of Materials, Ministry of Education, Shandong University, Jinan 250061, China

**Keywords:** performance-optimized multi-axial geogrid, pre-punched structure design, tensile forming, finite element simulation

## Abstract

In order to solve the problem of low transverse tensile strength of triaxial geogrid, a kind of performance-optimized multi-axial geogrid (POMG) that can bear larger transverse loads was designed. Firstly, the forming equipment and process of POMG are designed. Secondly, through the test of formability and mechanical properties, the POMG with good formability and mechanical properties is obtained, and the average tensile strength of POMG with circular and semicircular holes is the highest, reaching more than 16 KN/m. Finally, the feasibility of the process is further verified by numerical simulation, and the shape distribution and stress-strain law of POMG during the forming process are obtained, which provides further guidance for the actual production.

## 1. Introduction

Geogrid is one of the most important geosynthetics. It is a two-dimensional or three-dimensional structure formed by raw materials such as polypropylene (PP), high-density polyethylene (HDPE), or polyvinyl chloride (PVC). Geogrid has high tensile strength, low creep, corrosion resistance, aging resistance, and low quality, which can greatly improve the interlocking and occlusal ability of the reinforced bearing surface, and enhance the stability of soil and rock, so it is widely used in the fields of pavement reinforcement, slope protection and so on [1,2,3].

According to the geometric configuration, civil geogrid can be divided into uniaxial geogrid, biaxial geogrid, multi-axial geogrid. Uniaxial geogrid is obtained by uniaxial tension of punched polypropylene or high-density polyethylene sheet, which can provide higher strength in a single direction. Biaxial geogrid and multi-axial geogrid are obtained by twice stretching the punched polypropylene sheet. Biaxial geogrid can bear load in two main directions, while multi-directional geogrid can provide more quasi-isotropic load capacity in multiple directions.

**Figure 1:**
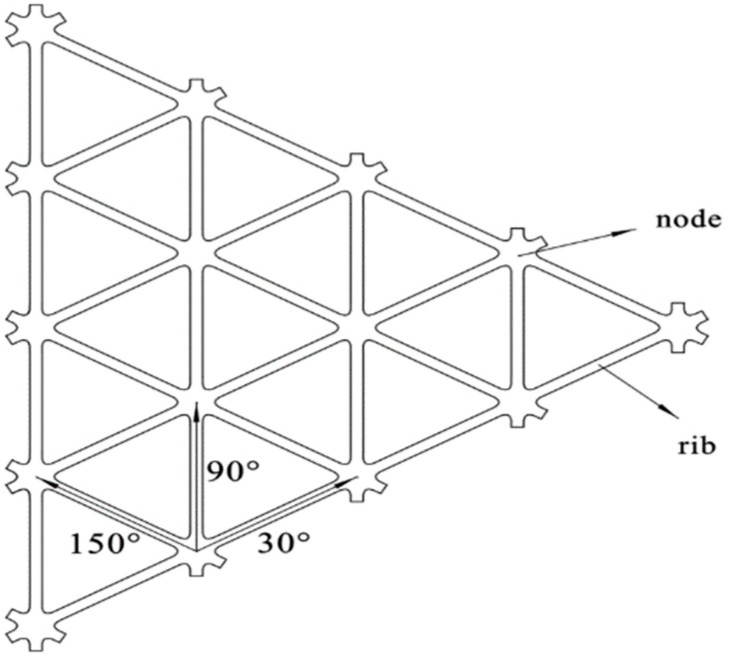
Basic structure of triaxial geogrid.

Geogrid is a multi-reinforced structure, and the basic element is called tendon, which includes two parts: ribs and codes. The basic structure of the tri-axial geogrid is shown in Figure 1. In the forming process, the rib has a large deformation along the tensile direction, and the code part basically does not participate in the deformation. The transition zone is the part connecting the ribs and codes, and the thickness of the geogrid in the transition zone has changed greatly.

The production process of geogrid mainly includes material extrusion, rolling, plate pre-punching, one or two stretching at high temperature, cooling, and setting at room temperature. The uniaxial geogrid is stretched only once, and the tension ratio is 8 to 10 times. The biaxial geogrid needs to be stretched longitudinally at a high temperature, and then stretched transversely at a high temperature. The tensile ratio of biaxial geogrid is generally 3–5 times [4]. The production process of multi-directional geogrid is similar to a biaxial geogrid, but the requirement of process parameters is stricter because the pre-punching structure of multi-axial geogrid is more complex and the interaction between ribs in the forming process is greater.

For plastic geogrid, the key factors affecting its performance include material, pre-punched plate, and process parameters.

The materials of geogrid are generally high-density polyethylene (HDPE), industrial polypropylene (PP), and polyethylene terephthalate (PET). Among them, industrial PP is generally used to produce multi-axial geogrid. Some properties can be improved by adding additives to polypropylene. For example, experiments show that the injection of nanofibers into polypropylene increases the tensile modulus and yield strength, but reduces the ductility [5], the addition of about 2% carbon black can inhibit the photoaging of the geogrid [6], and the addition of antioxidants can effectively prevent the oxidation reaction of the polymer [7].

The process parameters of the pre-punched plate mainly include the thickness of the sheet and the size, shape, and spacing of the pre-punched hole, which determine the structure of the geogrid to a certain extent. Zheng et al. [8] discovered the transverse distance between circular pre-punched holes directly affects the deformation and tensile fracture behavior of geogrids, and that too-small hole diameter results in the limitation of the formation and failure of geogrids. Ren et al. [9] found that under the condition of meeting the forming requirements, the smaller the diameter-to-distance ratio, the better the tensile strength, nominal elongation, and performance utilization of biaxial geogrid. When the longitudinal spacing is 1% larger than the transverse spacing, the material performance utilization factor has reached the maximum.

Tensile process parameters impact the tensile behaviors of geogrid [10]. Tensile temperature can affect the rheological properties of materials and the stress and strain distribution during the tensile process. The tensile temperature of industrial PP geogrid is generally 383–413 K. The experimental results show that the tensile stress-strain response of polypropylene strongly depends on strain rate and test temperature [11]. In general, reducing the tensile speed and increasing the tensile temperature can impact the stress-strain response similarly [12]. The stretch ratio determines the size and shape of the geogrid. When the stretch ratio is too small, geogrid will have insufficient tensile and poor mechanical properties. Conversely, if the tensile ratio is too large, geogrid will break. The stretching ratio of the multi-axis geogrid is generally about three.

In the process of stretching, the punched plate is stretched in a closed heating space, so the deformation behavior and the forming quality are difficult to observe. The wide application of the finite element method can provide detailed information on deformation and provide guidance for practical production. Sweeney et al. [13] have proved a theory for the large deformation modeling of polymers considering necking instability; a finite element analysis scheme was established and successfully applied to polypropylene sheets at 150 °C for both uniaxial and biaxial stretch. Based on the hyperelastic constitutive model, the final shape of the PP geogrid is predicted by Caton-Rose et al. [14], which verifies the feasibility of ABAQUS software to simulate the tensile process of unidirectional geogrid. Mimaroglu et al. [15] have reported the numerical study of geometric and material instability in uniaxially drawn polymers. They employed the finite element technique to model the uniaxial drawing behavior of polymers and obtained the relationship between the neck profile, neck propagation, and uniaxial stress-strain behavior of polymers. Zheng et al. [8] studied the deformation behavior of isotactic polypropylene in the manufacturing process of uniaxial geogrids by experimental and numerical methods. Ren et al. [16] established a triaxial tensile model to simulate the forming process of triaxial geogrid, through experimental verification, accurate results of forming law can be obtained. Therefore, the finite element method can save expensive experimental work and has always been a valuable product development tool in the manufacturing process of geogrid. 

Compared with uniaxial geogrids and biaxial geogrids, triaxial geogrid or multi-axial geogrid has more uniform mechanical properties in multiple directions. The performance and application of triaxial geogrid have been studied by many scholars. Zhang et al. [17] studied the tensile strength of biaxial geogrid and triaxial geogrid relative to the rib direction in different loading directions, and the results showed that compared with biaxial geogrid, triaxial geogrid could provide an almost uniform tensile strength in all loading directions. Dong Y et al. [18] studied the mechanical responses of biaxial and multi-axial geogrid under tension based on the finite element method, and the results showed that the distribution of the tensile strength and stiffness of the multi-axial geogrid was more uniform and could effectively withstand multi-axial loads. In addition, in the application of geogrid, multi-axial geogrid has a better reinforcement effect. Zhou et al. [19] discussed the influence of the arrangement of reinforcement in the basic unit of geogrid on the strength of reinforced sand through a triaxial shear test. The results show that the reinforcement effect of multi-directional geogrid is the best. Ma et al. [20] found that triaxial geogrid reinforcement is more economical and effective at reducing the uneven settlement and lateral displacement of a filled embankment.

However, in the actual application environment, we find that the triaxial geogrid is more prone to failure in the transverse direction under the same load. The study of Chen [21] also confirmed that the triaxial geogrid in the transverse direction has experienced a larger displacement than the longitudinal one, and the displacement difference increases significantly with the increase of normal stress. Multi-axial geogrid is expected to solve this problem. At present, the multi-axial geogrid mainly uses different small elements to refine the basic elements of the traditional geogrid to obtain different strengthening structures. Andrew Curson et al. [22] designed a multi-axial geogrid with hexagonal, trapezoidal, and triangular repeating elements, each outer hexagon extends inward to support and surrounds the smaller inner hexagon, thus forming multiple trapezoidal openings and a single hexagonal opening. The directional strands and partial directional connections of the outer hexagon form several linear strong axes, which extend continuously in the whole geogrid and form additional triangular openings. Robinson, WJ et al. [23] measured the response data of the roadbed strengthened by these two kinds of geogrid under traffic load. The results show that the geogrid redistributes higher pressure in the accumulation layer and effectively changes the variation of the measured stress profile with depth.

Therefore, combined with the above research, a performance-optimized multi-axial geogrid (POMG) was designed from the raw materials, pre-flushed plate structure, and forming process. The feasibility of the design and process is verified by experiment and numerical simulation, and the tensile process of POMG is analyzed in detail.

## 2. Design of Performance Optimized Multi-Axial Geogrid Structure

Based on the triaxial geogrid, the idea of a multi-axial geogrid structure is proposed.

**Figure 2:**
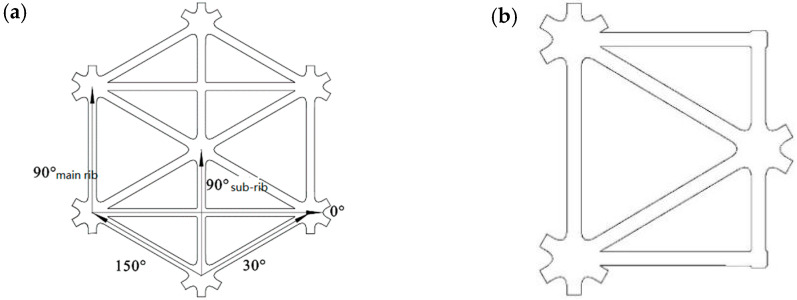
Structure of performance optimized multi-axial geogrid. (**a**) Rib naming method (**b**) Basic structural unit.

Figure 2 shows the design of a novel performance-optimized multi-axial geogrid (POMG). All the rectangular elements form a network structure, and each rectangular element includes a central triangular hole and a side triangular hole, and the central triangular hole is arranged in pairs and distributed along the horizontal direction. A pair of side triangular holes are respectively arranged on the upper side and the lower side of the central triangular holes, arranged in pairs.

While retaining the triangular structure of the triaxial geogrid, to improve the transverse strength of the triaxial geogrid, transverse ribs are arranged. It is characterized by a multi-rib porous structure, which not only has strong vertical and horizontal tensile strength, but can also carry a large load in the direction of 30° and 150°.

## 3. Tensile Forming and Fracture Test of Performance Optimized Multi-Axial Geogrid

Pre-punched plate and forming processes are the key factors affecting the structure and performance of the geogrid. In this section, based on the structure of POMG, four schemes of a pre-punched plate are proposed, their effects on the formability of POMG are studied through experiments, and the mechanical properties are obtained by tests, to obtain the better scheme of the pre-punched plate.

### 3.1. Experimental Design

#### 3.1.1. Tensile Forming Process and Machines

The industrial PP plates used in this experiment were provided by Feicheng Lianyi Engineering Plastics Co., Ltd., Taian, Shandong, China. The forming test is divided into two steps including the production of the pre-punched plate and high-temperature tensile forming.

**Figure 3:**
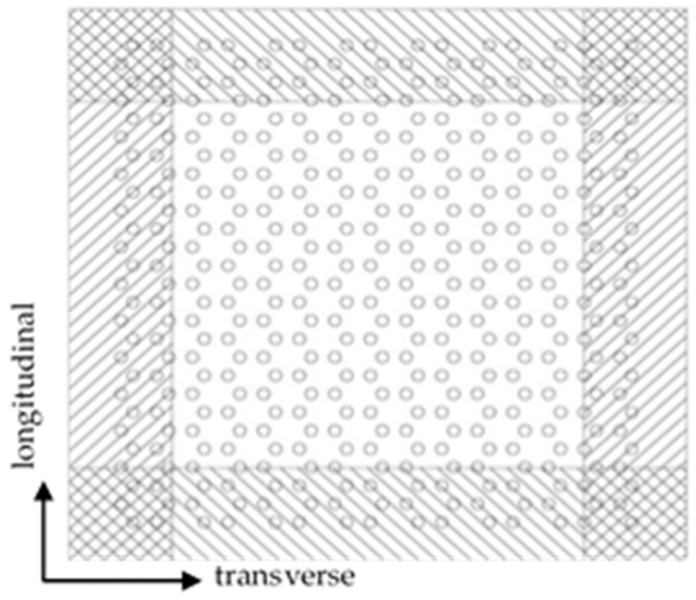
Pre-punched plate of performance-optimized multi-axial geogrid.

The first step is to press the pre-punched plate. In this step, firstly, the industrial polypropylene sheet is cut to make a plate, as shown in Figure 3, and pre-punched plate is square; the thickness of the plate is 4 mm, with a length and width of 150 mm, and a clamping area of 25 mm is reserved on each side. Then the laser cutting machine is used to punch different holes according to the pre-designed path. The accuracy of the laser-cutting machine SF1390 used in the experiment is 0.01 mm, which can meet the precision requirements of the 5A sample and punch.

The second step is to conduct high-temperature tensile forming on the pre-punched plate. Through experiments [11], it is determined that the optimum temperature of industrial PP material for geogrid preparation is 393 K and the tensile speed is 100 mm/min. In this chapter, the geogrid tensile forming machine used in the experiment mainly includes a longitudinal tensile system, a transverse tensile system, a temperature control system, and a control center, which can realize the test of the tensile forming process of different geogrids. The stretch process conditions used are shown in Table 1, after heating and heat preservation, the pre-punched plate is first stretched longitudinally, then transversely. After stretching, the distance between two adjacent codes of transverse ribs is 30 mm, and the distance between two adjacent codes of longitudinal main ribs is 34.6 mm.

#### 3.1.2. Mechanical Property Indexes of the Tensile Fracture Test

The mechanical properties and product naming rules of uniaxial and biaxial geogrids are specified in the Chinese national standard GB/T 17689-2008 [24] and the American national standard ASTM D 6637 [25]. For the whole forming geogrid, the color should be black, uniform in color, the appearance should be free of damage or rupture, the size and shape of the mesh should be uniform, and the carbon black content should be greater than or equal to 2%. The relevant mechanical properties include tensile strength, nominal tensile strength, nominal elongation, and creep, etc. The mechanical properties of geogrid can be tested by single rib method or multi-rib method.

Compared with the uniaxial and biaxial stretching geogrid, multi-axial geogrid lacks the corresponding relationship between product specifications and specific parameters. According to the characteristics of the multi-axial geogrid, tensile strength and elongation at break are selected as the mechanical performance indicators of POMG. Furthermore, the concept of multi-axial average tensile strength is proposed.

**Figure 4:**
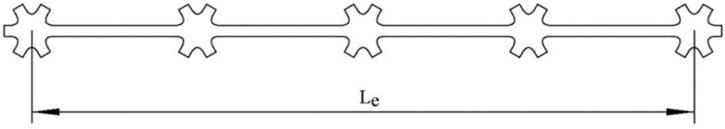
Sampling method for tensile test at room temperature.

The POMG was sampled by single rib method at room temperature, and the sample was tested by an electronic universal testing machine at room temperature. Sampling should select a part with evenly distributed nodes and ribs. The length of the sample should include at least two basic elements, and the effective length should not be less than 100 mm. The sampling method is shown in Figure 4.

In Figure 4, *L_e_* is the effective length of sampling, and is the distance between the center positions of nodes at both ends of sampling. In this experiment, *L_e_* is not less than 100 mm. During the clamping process, it should be ensured that the clamping fixture clamps the node part at both ends of the sample, and ensure that the tensile direction is parallel to the specimen holding direction. The tensile strength, elongation at break, and multi-axial average tensile strength were obtained by the room temperature tensile test, which were defined and calculated as follows.

1. Tensile strength is an important indicator to evaluate the performance of a geogrid, and its calculation method is as follows.

(1) $$F=\frac{f\times N}{n\cdot L_{e}}$$

*F* is the tensile strength, the unit is KN/m; *f* is the tension value of the sample, the unit is KN; *N* is the rib number of the sample in the direction to be measured; *n* is the number of bars in the direction not to be measured, *n* is no less than 2 for the multi-rib method, and equal to 1 for the single-rib method; *L_e_* is the effective width of the sample in the direction to be measured.

2. Elongation at break represents the ductility of geogrid under tension, and its calculation method is as follows.

(2) $$\delta =\frac{\Delta G}{G_{0}}$$

Δ*G* represents the displacement of the fixture along the tensile direction when the fracture occurs, and the unit is mm; *G*_0_ represents the distance between the clamping points of the fixture under the condition of pre-tension.

3. The multi-axial average tensile strength can be used to compare the average of the upward tensile strength of all sides of the multidirectional geogrid, and then measure the comprehensive mechanical properties of the multidirectional geogrid. The calculation method is as follows.

(3) $$\bar{F}=\frac{\sum_{i=1}^{s}F_{i}}{s}$$

If a geogrid basic unit contains s bars, the tensile strength in each rib *F*_1_, *F*_2_, *F*_3_, …, *F_S_* should be obtained first, and then the tensile strength in each direction was summed and the average value was calculated.

### 3.2. The Result of the Tensile Forming Experiment

**Figure 5:**
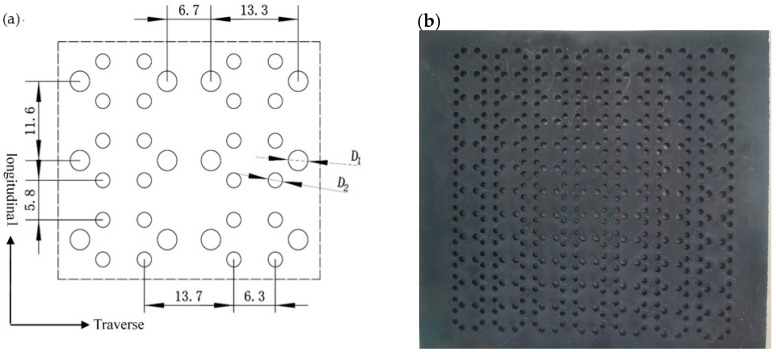
Hole arrangement of circular-circular pre-punched plate. (**a**) Plane diagram. (**b**) Physical drawing.

To obtain POMG with good performance, different pre-punched plate schemes were tried. Compared with other punches, round punches are easy to process and easy to mold debugging and maintenance, the pre-punch of POMG should be preferred under the premise of not affecting the molding and mechanical properties. So circular holes are tested first, predictably from the basic structure of Figure 2b, the pre-punched plate should be designed with circular holes of two different sizes. With reference to our work on the hole size and design of the triaxial geogrid [19], the Abaqus software is used in pre-design for hole size and arrangement. As shown in Figure 5, the diameter of the large circle is D_1_ = 3.2 mm, and the diameter of the small circle is D_2_ = 2.25 mm.

**Figure 6:**
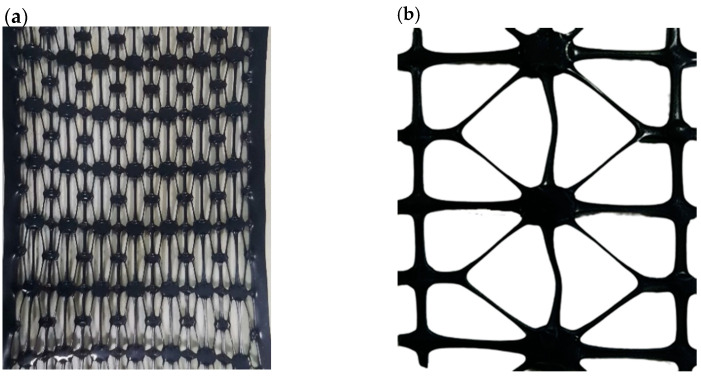
Performance-optimized multi-axial geogrid of circular-circular pre-punched holes. (**a**) Pre-punched plate after longitudinal drawing. (**b**) POMG formed fully.

The stretch process parameters in Table 1 are used to stretch the circular pre-punched plate at high temperature, and POMG with circular-circular pre-punched holes is obtained, its shapes after drawing once and drawing twice are shown in Figure 6.

As seen in Figure 6, the 90° main rib appears to bend to a certain extent, which is due to the large inward shrinkage force during the transverse stretching process. In addition, the nodes not connected with the transverse ribs have transverse deformation, which indicates that the small circular hole has not achieved the expected forming effect.

**Figure 7:**
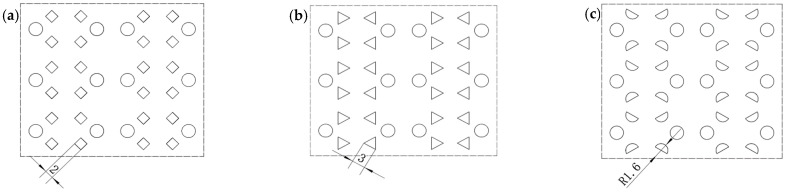
Hole layout scheme of other pre-punched holes. (**a**) Circular-diamond holes, (**b**) Circular-triangle holes, (**c**) Circular-semicircular holes.

Therefore, according to the principle of equal area, the small round holes need to be improved. There are three alternatives: diamond hole, triangle hole, and semicircular hole, and their same area is 4 mm^2^. The pre-punched hole arrangement is shown in Figure 7.

In Figure 7, the circular diameters are all 3.2 mm. In Figure 7a, the length of the rhombus side is 2 mm, and the angle between adjacent sides is 90°. In Figure 7b, the side length of an equilateral triangle is 3 mm; in Figure 7c, the semicircle radius is 1.6 mm.

**Figure 8:**
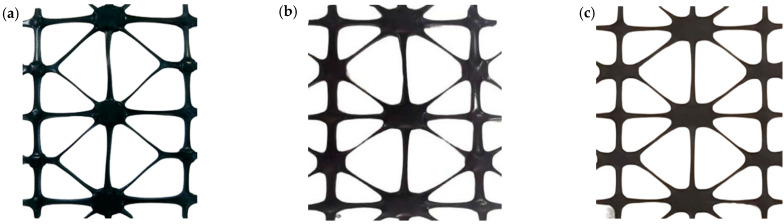
Performance-optimized multi-axial geogrid of other pre-punched holes. (**a**) Circular-diamond holes, (**b**) Circular-triangle holes, (**c**) Circular-semicircular holes.

The above three pre-punched holes schemes were subjected to high-temperature tensile tests using the tensile process parameters in Table 1. Select POMG have stable forming from multi-batch results; the results are shown in Figure 8.

According to Figure 8, when the pre-punched hole scheme of the circular-triangular hole and the circular-semi-circular hole is adopted, the nodes on the 90° sub rid do not deform along the transverse direction and the rid is straight. So, this means that the formability of circular-semicircular holes is better than others.

### 3.3. The Result of the Room Temperature Tensile Test

**Figure 9:**
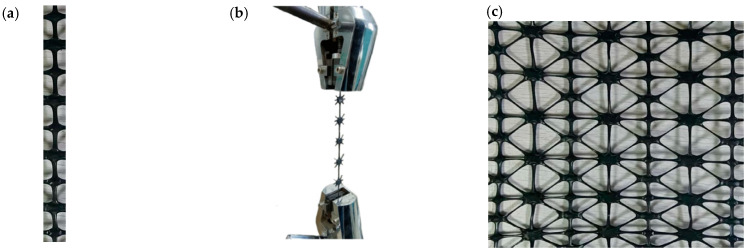
Test method for mechanical properties of single rib method. (**a**) Simple (90° sub-rib). (**b**) Single rib clamping method. (**c**) POMG with circular and semicircular holes.

The tensile strength, elongation at break, and multi-axial average tensile strength were obtained by using the electronic universal testing machine. Take the 90° sub-rib of POMG with circular and semicircular holes as an example; the sample and clamping methods are shown in Figure 9. Five ribs named in Figure 2a are selected for the samples.

According to GB/T 17689-2008 [24], 20% of the distance between sample fixtures per minute is taken as the drawing speed. This test was carried out at the same speed, at least four times for each group of tests. The tensile strength and elongation at break were counted, and the multi-axial average tensile strength was calculated according to the tensile strength of each side upward.

#### 3.3.1. Tensile Strength

The tensile strength of POMG under different schemes of pre-punched holes are shown in Table 2.

**Figure 10:**
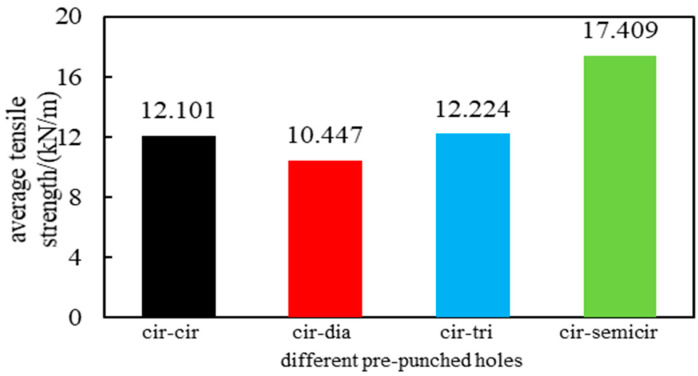
Average tensile strength of performance-optimized multi-axial geogrid with different holes.

As seen in Table 2, in the 0° direction, the POMG with circular and circular holes has the maximum tensile strength. Except for the 0° direction, the POMG formed by circular and semi-circular schemes is much stronger than others in all directions. For further comprehensive tensile properties of POMG, the average tensile strength of POMG with different pre-punched schemes was calculated, and the results were shown in Figure 10.

In Figure 10, the average tensile strength of the POMG with circular and semi-circular holes is the largest, which is 17.4 KN/m. The average tensile strength of POMG with circular-diamond holes is the smallest. Combined with Table 2, the tensile strength of POMG with circular-semicircle holes is far more than that of other pre-punched schemes. Therefore, if only considering the tensile strength, a circular-semicircular scheme should be chosen.

#### 3.3.2. Elongation at Break

The multi-axial geogrid with good mechanical properties should have a relatively large tensile strength and a relatively small elongation at fracture. For biaxial geogrids, the nominal elongation of longitudinal ribs should not be more than 15%, and the transverse ribs should not be more than 13% [24]. Since POMG have a rectangular structure of biaxial geogrids, the fracture elongation of the geogrids should meet the standard of biaxial geogrids. The fracture elongation of the 90° main rib and the 90° sub rib should not be more than 15%, and the 0° rib should not be more than 13%. Through the room temperature tensile test, the elongation at the break of the POMG was obtained, as shown in Table 3.

As seen from Table 3, elongation at the break of POMG with the circular and semicircular scheme is the smallest in the 0° direction, and elongation at the break of POMG with the circular and semicircular scheme in the 30° and 150° directions is the largest. In addition, as shown in the data in bold in Table 4, the elongation at the break of the 90° sub-rib and the 90° main rib of POMG formed by circular-circular, circular-diamond, and circular-semicircular schemes all exceeded the prescribed nominal elongation. This means a conclusion that POMG formed by circular and semicircular schemes has a great advantage in the 90° direction.

#### 3.3.3. Effective Tensile Strength

When the fracture elongation of POMG is greater than the prescribed nominal elongation, it is necessary to evaluate the tensile strength when the elongation is the nominal elongation, that is, the effective tensile strength. Therefore, it is necessary to compare the effective tensile strength of POMG with different schemes, then determine their advantages and disadvantages.

The effective tensile strength of POMG with different pre-punched hole schemes was calculated based on the data of the room temperature tensile test, as shown in Table 4.

**Figure 11:**
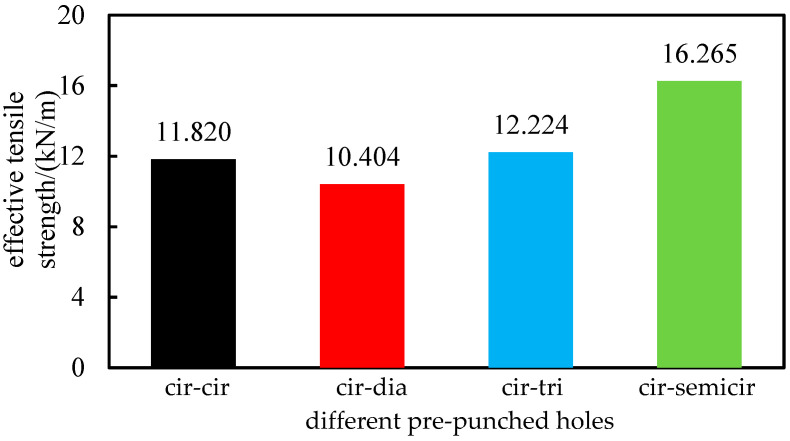
Average of effective tensile strength of performance-optimized multi-axial geogrid with different pre-punched holes.

By comparing Table 2 and Table 4, when the nominal elongation is reached, the tensile strength of the 90° main and sub ribs of POMG with circular and semicircular holes is still greater than that of other schemes. According to the definition of effective tensile strength, if the elongation at the break of the geogrid is less than the nominal elongation, the actual tensile strength is the effective tensile strength. The average effective tensile strength of POMG with different pre-punch holes is shown in Figure 11.

As shown in Figure 11, the average effective tensile strength of POMG with circular and semicircular pre-punched holes is the largest.

Combining Section 3.2 and Section 3.3, in conclusion, the POMG with circular and semicircular pre-punched holes can not only achieve better formability but can also obtain optimal mechanical properties. In the actual production of POMG, the pre-punched method of large circular holes and semicircular holes should be adopted.

## 4. Simulation Study on the Forming of Performance Optimized Multi-Axial Geogrid

In the third part, it is concluded that the forming and mechanical properties of POMG are the best when circular and semicircular holes are adopted. The forming process of POMG is complicated, to further study the forming of POMG, this section takes the POMG with circular and semicircular holes as the object and uses Abaqus CAE software to analyze the heat transfer, high-temperature stretching process, and forming results. Through analysis, the distribution law of thickness, width, stress, and strain of POMG is obtained. These works will provide guidance to produce POMG.

### 4.1. Establishment of Material Constitutive Mode

The algorithms in Abaqus CAE include an explicit algorithm (central difference method) and an implicit algorithm (Newmark method). Compared with an implicit algorithm, an explicit algorithm is more suitable for dynamic analysis, without equilibrium iteration, faster, and easier to converge.

Therefore, the explicit algorithm should be selected for the numerical simulation analysis of the tensile forming of geogrid.

The interior of industrial polypropylene is composed of the crystal region and the amorphous region. Under the action of tensile force at high temperatures, the crimped chain segment of industrial polypropylene extends and the polymer is deformed. The molecular mechanism of large deformation of materials is mainly the segment motion of polymers. After the yield point, the frozen segments of the polymer begin to move under the action of a large external force, and the extension of the polymer chain provides a large deformation of the material. If the temperature is raised above the glass transition temperature and the external force is unloaded, the deformation can be restored; if the temperature is reduced to below the glass transition temperature, the deformation will be retained. The superelasticity of the material model in Abaqus CAE can be used to numerically analyze the tensile process of various glassy or superplastic polymers [26].

The Marlow constitutive model in the hyperelastic model of Abaqus CAE can accurately fit the stress-strain curve with yield point and accurately simulate the yield-necking-hardening process of glassy polymer.

Marlow’s constitutive model uses strain potential energy *U* to describe the stress-strain relationship of hyperelastic materials. The strain potential energy defines the strain energy stored per unit volume of the material as a function of the strain at that point. The strain potential energy function of the material in the Marlow model can be expressed as a Formula (4).

(4) $$U=U_{dev}\left(\bar{I_{1}}\right)+U_{vol}\left(J_{el}\right)$$

*U* is the unit strain potential energy, *U_dev_* is the stress-strain potential energy, *U_vol_* is the volumetric strain potential energy, *J_el_* is the elastic volume ratio, and *I*_1_ is the first order partial strain invariant, which can be expressed by Formula (5).

(5) $$\bar{I_{1}}={\bar{\lambda }}_{1}{}^{2}+{\bar{\lambda }}_{2}{}^{2}+{\bar{\lambda }}_{3}{}^{2}$$

where *λ*_1_ is the drawing ratio in the main direction. In Formula (4), the stress part energy is determined by uniaxial, biaxial, or plane test data, and the volume part energy is determined by volume test data [9].

Abaqus CAE uses the least square method [26] to obtain the material strain potential energy function from the material stress-strain data. The simulation assumes that the industrial PP is an isotropic material, and the stress-strain data are obtained by uniaxial tensile test and imported into the analysis. According to the Chinese national standard GB/T 1040.2-2006 [24], Industrial PP sheets were sampled and tested at high temperatures. The tensile rate was set at 100 mm/min and the stretching ratio was set at eight times, and the nominal stress-straincurves of the material were obtained at 373 K, 383 K, 393 K, 403 K, and 413 K, This numerical simulation is fitted by the stress-strain curve of industrial PP.

**Figure 12:**
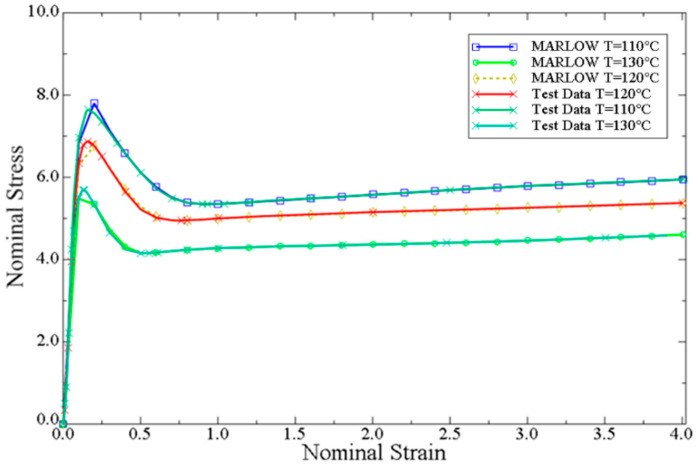
Fitting of stress-strain test values and Marlow curves at different temperatures.

The fitting situation is shown in Figure 12.

As seen from Figure 12, there is little deviation between the Marlow curves and the test value in the elastic stage, and when the yield point is reached, the Marlow curves are basically consistent with the test values. Therefore, the Marlow curve can better describe the stress-strain behavior of industrial polypropylene during tension at 383 K to 403 K, so the Marlow constitutive model is selected in this numerical simulation.

**Figure 13:**
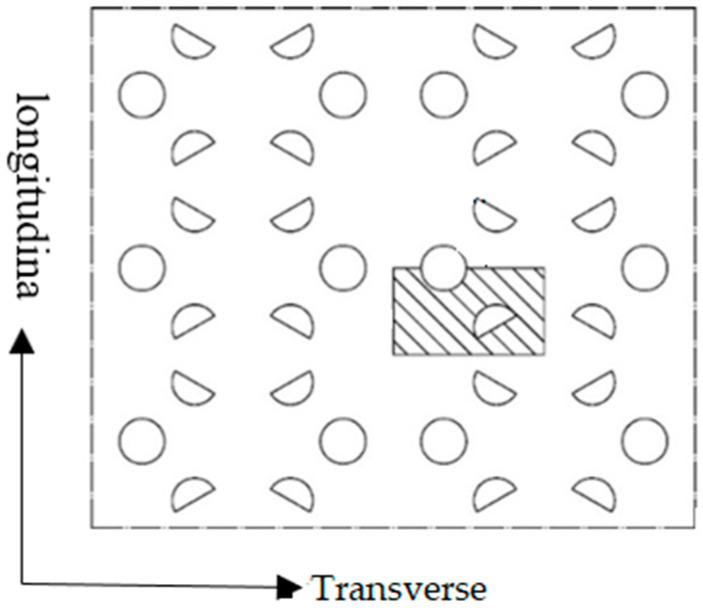
Basic unit of the simulation of performance-optimized multi-axial geogrid.

In this section, the shaded area in Figure 13 will be used as the basic unit for simulation use. The distance in the longitudinal direction of the simulation unit is 10 mm, and the distance in the transverse direction is 5.8 mm. The thickness is 4 mm. The pre-punched arrangement is the same as shown in Figure 5.

**Figure 14:**
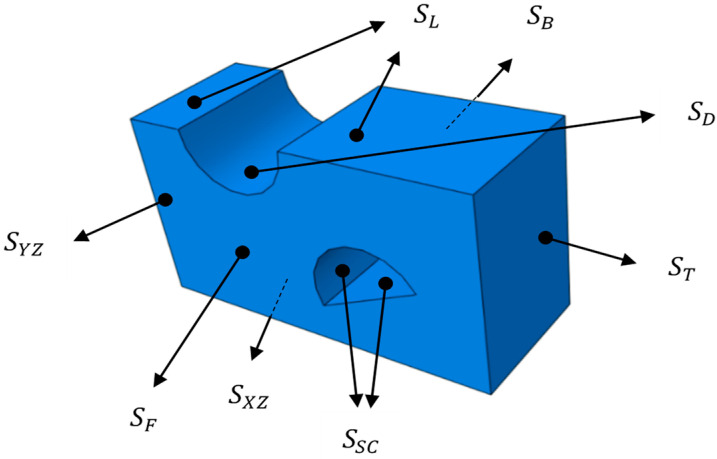
Loading surface with boundary conditions of the basic unit of the simulation.

After setting the basic simulation unit, as shown in Figure 14, the basic simulation unit is modeled and the loading surface is named. Heat transfer simulation and tensile simulation are carried out.

### 4.2. Heat Transfer Simulation of Composite Multi-Axial Geogrid

The industrial polypropylene sheet at room temperature does not have strong tensile properties, and the stretch ability is small. Therefore, the pp plate needs to be preheated at a high temperature for a certain time before stretching. In this section, through the heat transfer simulation of the basic unit with circular and semi-circular holes, the temperature distribution law is obtained, which is a predefined field before stretching.

In the Initial step, boundary conditions were set to define the specular symmetry of the model with *S_YZ_* and *S_XZ_* as symmetry surfaces. Set the initial temperature to room temperature 293 K. In the heat transfer step, the heat transfer surface of the model was set as *S_F_*, *S_B_*, *S_D_*, and *S_SC_*, and the ambient temperature in the oven was 393 K, high temperature holding time is 5 min., and the temperature and time are same as those in Table 1. The thermal conductivity of industrial PP is set to 0.24 [27]., the eight-node linear heat transfer hexahedral element DC3D8 was set, and the mesh size was 0.10 mm [28].

**Figure 15:**
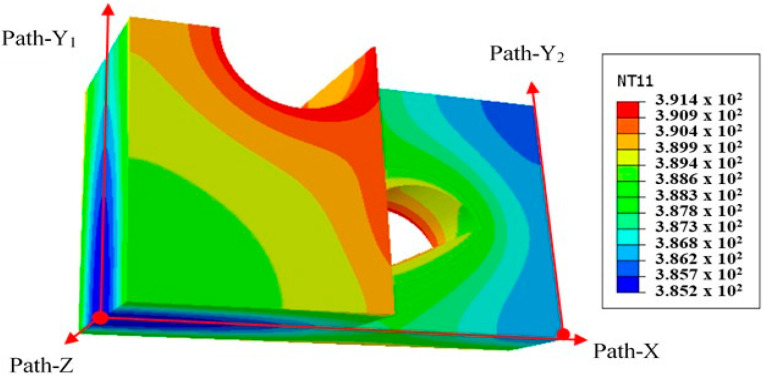
Heat transfer simulation results and path setting.

The heat transfer simulation results and path setting of the circular and semi-circular pre-punched model are shown in Figure 15.

As seen in Figure 15, the highest temperature of the model occurs at the junction between the semi-circular and circular punching holes and the model surface, and the temperature can reach over 390 K, mainly due to the large contact surface between this area and the external environment and the high heat transfer efficiency. This part of the material is prone to deform first in the subsequent stretch process. The lowest temperature of the model occurs at a distance from the punching hole, about 385 K, this part tends to form nodes in the subsequent stretching process.

**Figure 16:**
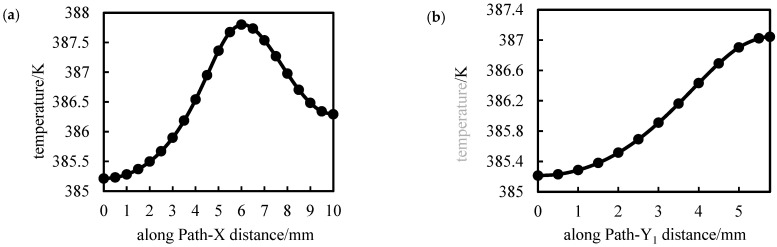

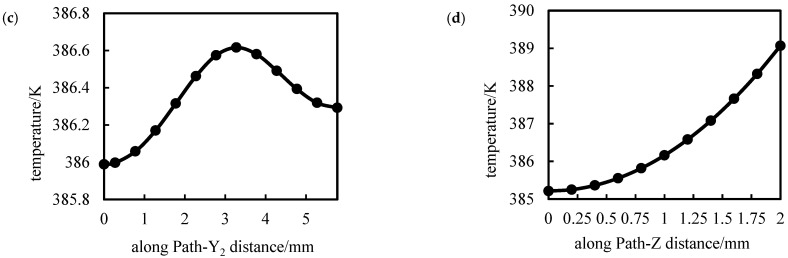
Temperature distribution along each path of performance-optimized multi-axial geogrid. (**a**) Path-X, (**b**) Path-Y_1_, (**c**) Path-Y_2_, (**d**) Path-Z.

The temperature distributions along path-X, path-Y1, PAth-Y2, and Path-Z paths were statistically analyzed, as shown in Figure 16.

As seen in Figure 16, along paths X and Y_2_, the temperature change trend is similar, first increasing and then decreasing, along paths Y_1_ and Z, the temperature increases with the distance. Combined with the simulation results in Figure 15, the temperature is related to the distance from the adoption point to the semicircular hole, and the temperature is the highest at the semicircular hole. The temperature between the edge core and the core of the model is lower.

By comparing the above figures, it is found that the heat transfer temperature of the region forming nodes in the subsequent stretching process is lower after completion, and the heat transfer temperature of the region forming ribs is higher.

### 4.3. Tensile Forming Simulation of Performance Optimized Multi-Axial Geogrid

In this section, the tensile forming simulation of POMG is carried out.

The simulated basic unit shown in Figure 14 is stretched longitudinally and laterally in turn, setting two “power, temperature-displacement, explicit” analysis steps, step-1 and Step-2, which respectively represent the longitudinal and transverse stretching processes, and impose boundary conditions on the partial loading surface shown in Figure 14.

In the initial step, the model is set to be symmetrical and specular symmetry, the boundary condition is inherited to the next step, and the heat transfer result file is imported as a predefined field.

In step-1, the model is set to be symmetrical and the load is applied to the plane so that the longitudinal displacement is 11.6 mm.

In step-2, the boundary condition set in Step-1 is deactivated, and the model is set to be symmetrical, and the load is applied to the plane so that the lateral displacement is 20 mm.

In this way, the stretch forming process of POMG can be simulated according to the process conditions in Table 1.

After the load and boundary conditions are set, an eight-node thermal coupled hexahedral element C3D8T was used for meshing. The mesh size was 0.1 mm, and each mesh should be consistent with the heat transfer step. The results are as follows.

#### 4.3.1. Overall Shape Analysis

**Figure 17:**
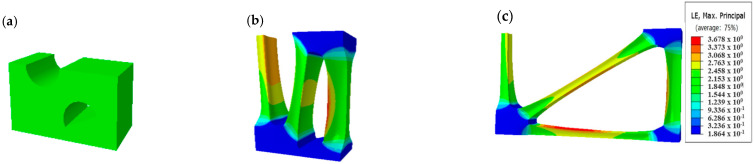
Strain contours of the stretching simulation of performance optimization multi-axial geogrid. (**a**) Initial shape, (**b**) Longitudinal stretch, (**c**) Transverse stretch.

Figure 17 shows the strain distribution obtained from the simulation of the stretch forming process of POMG with circular and semicircular pre-punched holes.

As seen in Figure 17, in the longitudinal stretch process, the main and sub ribs at 90° are basically formed, and 30° and 150° diagonal ribs are partially formed; 0° ribs are formed completely in a transverse stretch. In the process of stretching, the ribs deformed greatly, and the codes barely deformed.

**Figure 18:**
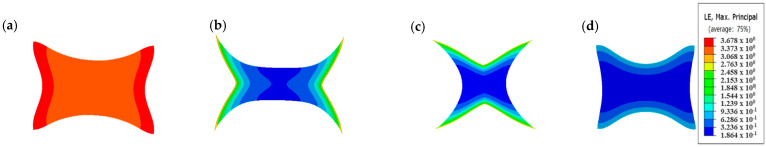
Cross sections of each rib after stretching of performance optimization multi-axial geogrid. (**a**) 90° main rib, (**b**) 90° sub-rib, (**c**) 0° rib, (**d**) 30° rib.

The cross-section of the rib after the transverse stretch of the simulated basic element is taken, and the change of the cross-sectional shape is shown in Figure 18.

As seen in Figure 18, the cross-section of each rib of the geogrid is thin in the middle and thick at both ends. This indicates that the temperature of the core material of each rib is lower than that of the surface material, and it is easy to deform first.

#### 4.3.2. Shape Analysis of Rib after Stretch

**Figure 19:**
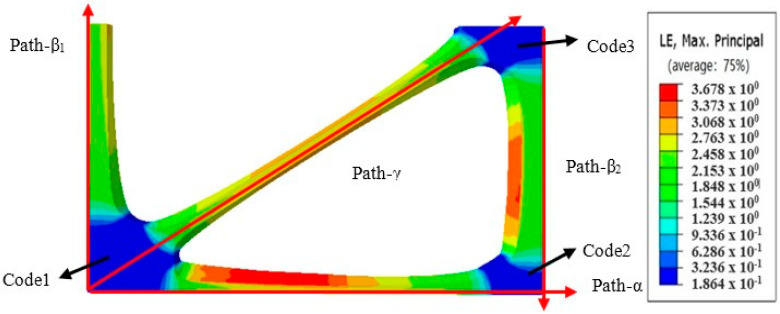
Path definition of performance optimization multi-axial geogrid.

The distribution rules of average thickness, average width, stress, and strain can be obtained more intuitively by defining the path of POMG after the completion of bidirectional stretching. The path definition is shown in Figure 19. Path-α is 0° rib, path-β1 is 90° main rib, path-β2 is sub-rib, and path-γ is 30° rib. Code 1 is formed by the intersection of 90° main rib, 30° rib, and 0° rib; code 2 is formed by the intersection of 0° rib and 90° sub-rib; code 3 is formed by the intersection of 30° rib and 90° sub-rib. Path-α was defined as the direction extending from code 1 to code 2, path-β1 was defined as the direction extending from 90° main rib, path-β2 was defined as the direction extending from code 3 to code 2, and path-γ was defined as the direction extending from code 1 to code 3 along 30° rib.

To verify the accuracy of the model, the length, minimum width, and thickness of path-α, path- β2, and path- γ of the actual geogrid and the model are taken. The results are as Table 5.

The comparison results show that the geometric shape of the geogrid is in good agreement with the numerical results and the experimental results. The error is partly due to the fluctuation of the material properties in the experiment.

**Figure 20:**
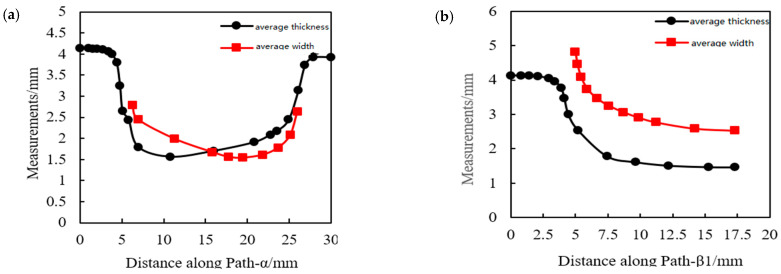

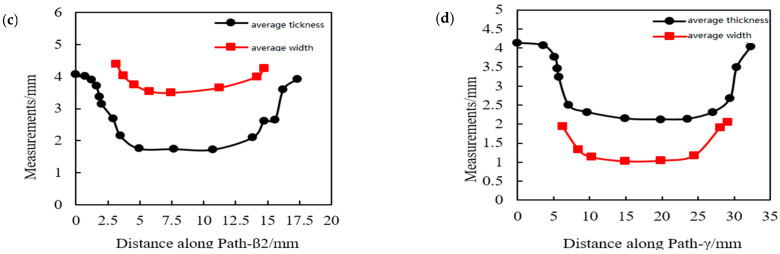
Distribution of average thickness and width of ribs along each path. (**a**) Path-α, (**b**) Path-β1, (**c**) Path-β2, and (**d**) Path-γ.

The path in Figure 19 can be used to collect the average thickness, width, and stress- strain of each rib after the completion of stretching. The average thickness and width of the ribs along each path after the stretch are shown in Figure 20.

The changing trend of average width and thickness in 0° rib, 30° rib, and 90° sub-rib are the same, which are larger at both ends, but smallest in the middle. The thickness of 0° rib is first less than its width and then greater than its width. The average width of 30° rib and 90° sub-rib is always greater than their thickness.

The width and thickness of the 90° main ribs decrease moderately with the distance, and the width is always greater than its thickness. Due to the fact that this is the same as the actual measured value.

To sum up, the average thickness of the 30° ribs is the largest and the average width is the smallest; the average thickness and width of all ribs decreases at first and then increases; the thickness at the codes is the largest, and the average thickness changes obviously in the transition zone.

#### 4.3.3. Stress-Strain Analysis

**Figure 21:**
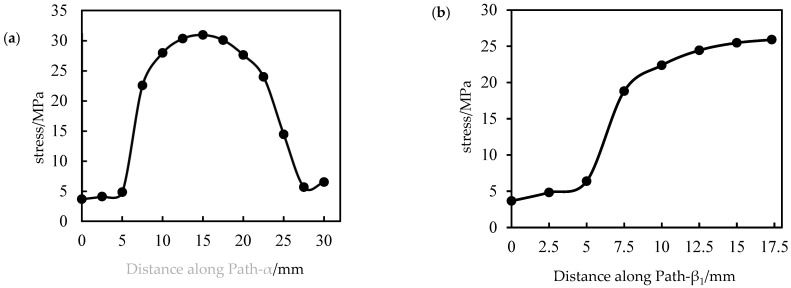

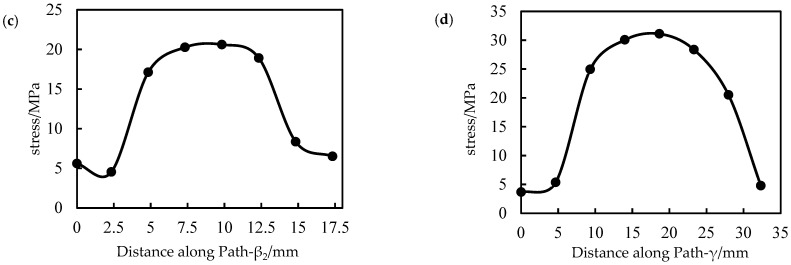
Distribution of stress of ribs along each path. (**a**) Path-α, (**b**) Path-β1, (**c**) Path-β2, (**d**) Path-γ.

The stress and strain data in the central area of ribs are collected along each path shown in Figure 18, and the stress distribution is shown in Figure 21.

As seen in Figure 21, the rib stress is much larger than the code and the transition area, the stress at the node is small, and the stress in the transition area changes significantly. This can explain that in the actual stretching process, the rib deforms the most, while the code hardly participates in the deformation. Considering the symmetry of the 90° main rib, the stress of each rib along the path direction shows a trend of increasing first and then decreasing.

**Figure 22:**
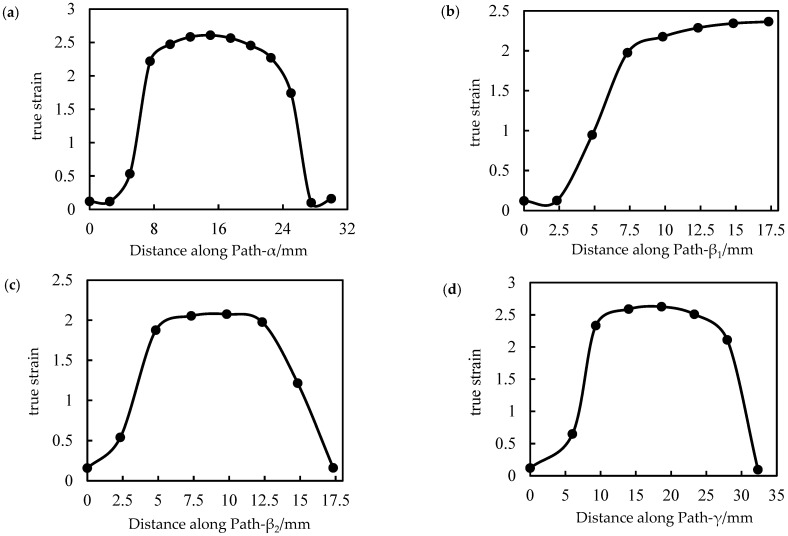
Distribution of true strain of ribs along each path. (**a**) 0° rib along Path-α, (**b**) 90° main rib along Path-β1, (**c**) 90° sub-bar along Path-β2, (**d**) oblique rib along Path-γ.

The logarithmic strain distribution in the central region of the ribs along each path is shown in Figure 22.

As seen in Figure 22, the real strain distribution is basically the same as the stress distribution. Along the central axis of each rib, the strain value of each code is small, and only a small degree of deformation occurs during the two stretching processes. The strain value of 90° sub-ribs is obviously lower than that of other ribs. The real strain value of 0° and 30° ribs is greater than 2.5, and obvious deformation occurs during the stretching process.

To sum up, during the stretching process, the rib mainly participates in the deformation, and its width and thickness change significantly relative to before stretching; in the transition area, geometry and stress-strain changes are the most drastic; the node basically does not participate in the deformation during the stretching process, and its thickness does not change much from that before the stretching.

## 5. Conclusions

To solve the problem of low transverse tensile strength of triaxial geogrid, through experiment and simulation, a kind of multi-axial geogrid with multi-rib porous structure and large tensile strength in multiple directions was designed. Through the research, the main conclusions are as follows.

1. Through the forming test, the ideal forming effect can be obtained when POMG uses a scheme of circular-triangle holes or circular-semicircular holes.
2. Through room temperature tensile test, the tensile strength of the geogrid is more than 40% higher than that of other schemes, except 0°, and the average effective tensile strength of the circular-semicircular composite scheme is more than 16 KN/m, which has a good application prospect.
3. Through the heat transfer simulation of POMG, it is found that the temperature is the highest in the area near the sheet surface and pre-punched holes after high-temperature heat preservation.
4. Through numerical simulation, it is found that the largest average thickness of each rib is at the code, the smallest at the rib, and the average width along the central axis decreases first and then increases. Comparing the width and thickness of different ribs, it is found that the thickness of 30° ribs is the largest and the width is the smallest.
5. Through numerical simulation, it is found that the stress and strain values in the rib along the central axis are larger than those in other regions, and the stress value in the central region of the joint formed by the intersection of 90° sub rib and 0° rib is larger than that in the edge region of the joint.

## Figures and Tables

**Table 1:** Stretching process parameter of performance-optimized multi-axial geogrid.

Parameters	Set Value
Material	Industrial PP
Stretch Ratio	3.0
Stretching Temperature	393 K (120 °C)
High-Temperature Holding Time	5 min
Stretching Speed	200 mm/min
Thickness of Plates	4.0 mm

**Table 2:** Tensile strength of performance-optimized multi-axial geogrid with different holes (KN/m).

	Direction	0°	30° Rib	90° Main Rib	90° Sub-Rib	150° Rib
Hole Schemes						
Circular-circular		**18.806**	6.960	15.936	11.396	7.407
Circular-diamond		15.149	4.950	15.716	11.640	4.782
Circular-triangle		17.256	6.963	16.292	12.180	8.428
Circular-semicircular		15.219	14.314	23.243	20.796	13.474

**Table 3:** Fracture elongation of performance-optimized multi-axial geogrid with different schemes (%).

	Direction	0°	30° Rib	90° Main Rib	90° Sub-Rib	150° Rib
Hole Schemes						
Circular-circular		12.02	8.35	11.59	26.21	9.31
Circular-diamond		11.01	6.58	11.78	17.49	5.96
Circular-triangle		11.52	8.32	11.94	11.16	8.00
Circular-semicircular		10.10	10.67	19.75	33.14	10.59

**Table 4:** Effective tensile strength of performance optimization multi-axial geogrid with deferent pre-punched holes (KN/m).

	Direction	90° Main Rib	90° Sub-Rib
Hole Schemes			
Circular-circular		11.59	9.991
Circular-diamond		11.78	11.422
Circular-semicircular		21.665	**16.655**

**Table 5:** Comparison of numerical and experimental dimensions.

	Direction	Experimental (mm)	Numerical (mm)	Absolute Error (%)
Hole Schemes				
α-length		32.5	30.2	8.1
α-width		1.74	1.8	3.4
α-thickness		1.58	1.7	7.5
β2-length		17.8	17.5	1.7
β2-width		3.12	3.6	15.3
β2-thickness		1.82	1.8	1.1
γ-length		34.2	32.5	4.9
γ-width		1.04	1.1	4.3
γ-thickness		2.10	2.2	4.7

## Data Availability

The data used to support the findings of this study are available from the corresponding author upon request.

## Abbreviation

POMG: performance-optimized multi-axial geogrid.
